# The relationship between body mass index and pain, disease activity, depression and anxiety in women with fibromyalgia

**DOI:** 10.7717/peerj.4917

**Published:** 2018-05-28

**Authors:** Burhan Fatih Koçyiğit, Ramazan Azim Okyay

**Affiliations:** 1 Department of Physical Medicine and Rehabilitation, Kahramanmaras Sütcü Imam University, Kahramanmaras, Turkey; 2 Department of Public Health, Kahramanmaras Sütcü Imam University, Kahramanmaras, Turkey

**Keywords:** Fibromyalgia, Body mass index, Disease activity, Depression, Anxiety, Obesity

## Abstract

**Background:**

Obesity is a possible factor which affects severity of symptoms and disease activity in fibromyalgia syndrome (FMS). The aim of our study was to determine the association between body mass index (BMI) and pain, tender point count (TPC), disease activity, anxiety and depression in patients with FMS.

**Methods:**

This was a descriptive study. A total of 124 female FMS patients between 18 and 55 years of age were enrolled. FMS patients were evaluated with visual analog scale (VAS), fibromyalgia impact questionnaire (FIQ), Hamilton anxiety scale (HAM-A) and Hamilton depression scale (HAM-D). Patients were divided into three groups according to BMI levels: normal weight, overweight and obese. Normal weight was defined as BMI 18.5–24.9, overweight as BMI 25.0–29.9 and obesity as BMI ≥ 30. We assessed the BMI status and its association with symptom severity in patients with FMS.

**Results:**

Significant differences were detected in VAS, TPC, FIQ and HAM-D among the groups (*p* < 0.05). There were no significant differences between the groups in HAM-A (*p* = 0.328). The highest scores were found in the obese group. Significant positive correlations were determined between BMI levels and VAS, TPC, FIQ and HAM-D (*r* = 0.277, *p* = 0.002; *r* = 0.384, *p* < 0.001; *r* = 0.292, *p* = 0.001; *r* = 0.357, *p* < 0.001).

**Discussion:**

Obese female FMS patients had higher levels of pain, TPC, disease activity and depression. BMI was significantly and positively correlated with clinical manifestations of FMS. Therefore, FMS treatment programs should include weight loss strategies.

## Introduction

Fibromyalgia syndrome (FMS) is a chronic rheumatic disease accompanied with widespread musculoskeletal pain, specific tender points, cognitive disturbance, depression, sleep disorders, irritable bowel syndrome, fatigue and morning stiffness (Wolfe et al., 1990). Variations in the clinical presentation of the disease may be an impediment for diagnosis and treatment strategy (Estévez-López et al., 2017). FMS is more common in females than in males (Yunus, 2001). FMS prevalence in female adults is between 2.4% and 6.8% (Marques et al., 2017). The exact etiopathogenesis of widespread pain in FMS patients is yet to be explained (Jahan et al., 2012). Deteriorations in the autonomic nervous and neuroendocrine systems, cytokines, genetic factors and environmental stressors may play a role in the etiopathogenesis (Bradley, 2009).

Obesity is a complex disorder, defined as excessive fat accumulation in the adipose tissues (de Araújo, Mota & Crispim, 2015). Obesity which has widely been accepted as an obstacle to pain management, is comorbid with back and neck pain, migraine and osteoarthritis (Janke, Collins & Kozak, 2007; Bond et al., 2011; Okifuji & Hare, 2015). In different studies, 62–73% of FMS patients have been reported to be overweight or obese (Kaartinen et al., 2000; Yunus, Arslan & Aldag, 2002; Neumann et al., 2008; Okifuji, Bradshaw & Olson, 2009). A higher body mass index (BMI) is a strong and an independent risk factor for future development of FMS (Mork, Vasseljen & Nilsen, 2010). Reduction in physical activity associated with widespread pain may cause an increased BMI (Ursini, Naty & Grembiale, 2011). Besides, there is a link between BMI and the clinical characteristics of FMS (Alciati et al., 2018). It has been reported that weight loss programs in overweight and obese FMS patients ameliorate the clinical symptoms (Benazzi & Akiskal, 2003; Shapiro, Anderson & Danoff-Burg, 2005). In addition, bariatric surgery in FMS patients has been shown to reduce the clinical symptoms (Hooper et al., 2007; Saber et al., 2008). Obesity and FMS have similar clinical features such as higher pain sensitivity, poorer sleep quality and lower quality of life (Yunus, Arslan & Aldag, 2002; Kim et al., 2012). Both obesity and FMS cause alterations in endocrine activity and opioid system that may influence the level of pain perception (Kawasaki et al., 2008). Additionally, increased BMI is associated with higher interleukin six levels which play an important role in inflammatory pathways and pain processing (Okifuji, Bradshaw & Olson, 2009). Obesity is a possible factor affecting severity of symptoms and disease activity in FMS patients. Thus, weight control may be a critical factor in the management of FMS symptomatology.

The primary aim of this study was to compare pain, tender point count (TPC), disease activity, anxiety and depression across different weight status categories in female FMS patients. Our secondary aim was to assess correlations between BMI and pain, TPC, disease activity, anxiety and depression in FMS patients.

## Materials and Methods

### Study design and participants

This was a descriptive study conducted between November 2017 and March 2018. A total of 157 female outpatients who applied to a physical medicine and rehabilitation polyclinic were evaluated. All the participants recruited in this study met the FMS criteria of American College of Rheumatology (ACR) 1990 (Wolfe et al., 1990). Other inclusion criteria were patients being between 18 and 55 years of age and being able to understand, read and write Turkish.

Exclusion criteria included a history of psychiatric disorders, immune deficiency, malignancy, diabetes mellitus, thyroid dysfunction, peripheral neuropathy, chronic inflammatory diseases, acute/chronic infection, pregnancy and breast-feeding.

Following the application of the exclusion criteria, a total of 124 FMS patients were enrolled in the study.

### Data sources and measurement

Patients filled in and signed a detailed questionnaire form that contained questions concerning patient age and, occupational, marital and educational status in addition to 10 cm visual analog scale (VAS), fibromyalgia impact questionnaire (FIQ), Hamilton depression scale (HAM-D) and Hamilton anxiety scale (HAM-A).

Visual analog scale is used to determine the severity of pain. Patients mark the severity of pain on a 10 cm long scale (0 = no pain, 10 = the most severe pain).

Further, the following 10 factors are measured by FIQ: physical function, feeling good, ability to work, having difficulties at work, anxiety, depression, pain, fatigue, morning fatigue and stiffness. The maximum score for each subdomain is 10, which makes the highest score 100 (Sarmer, Ergin & Yavuzer, 2000).

In addition, tender points were determined by applying digital pressure on 18 points as indicated in the ACR classification criteria (Wolfe et al., 1990). TPC data were evaluated as the number of counted positive tender point sites. The maximum score for TPC is 18.

The depression levels of the patients were assessed using HAM-D. In this scale, 14 points or more indicate depression. The anxiety levels of the patients were determined using HAM-A. It includes 14 items querying physical and mental indications. The existence and severity of measured items were evaluated by the interviewer (Hamilton, 1959).

We considered the World Health Organization criteria for the classification of weight status. BMI is calculated as weight in kilograms divided by the square of height in meters (kg/m^2^). A BMI level between 18.5 and 24.9 was suggestive of normal weight; between 25.0 and 29.9, overweight; and more than 30, obesity. Patients were grouped into three categories according to BMI levels: normal weight, overweight and obese.

### Ethical considerations

Our study was approved by Scientific Researches Ethics Committee of Kahramanmaras Sutcu Imam University (Decision date: 25.10.2017; Decision number: 02). Written informed consent was obtained from all participants and participation in the study was purely voluntary.

### Statistical analysis

Statistical analysis of data was performed with Statistical Package for Social Sciences (SPSS) for Windows version 20.0 package program (SPSS Inc., Chicago, IL, USA). All results are expressed as mean ± standard deviation, median (minimum–maximum), number and percentage. Normality of data distribution was evaluated with Shapiro–Wilk test. A Chi-squared test was performed to identify the differences in categorical variables between groups. Continuous variables were analyzed between independent groups with one-way analysis of variance or the Kruskal–Wallis test if variables were not normally distributed. Spearman correlation analysis was used to determine the correlations between BMI levels and VAS, TPC, FIQ, HAM-A and HAM-D scores. The statistical significance value was accepted as *p* < 0.05.

## Results

In this study, 124 female FMS patients were enrolled. Participants were divided into three groups: normal weight group, overweight group and obese group. Of the total, 43 patients (34.7%) were in the normal weight group, 40 (32.2%) were in the overweight group and 41(33.1%) were in the obese group. The mean ages in the normal weight, overweight and obese groups were 40.09 ± 7.26, 41.20 ± 7.65 and 43.31 ± 7.88 years, respectively (*p* = 0.148). The mean BMI levels were 23.21 ± 1.24 in the normal weight group, 27.92 ± 1.17 in the overweight group and 33.68 ± 3.38 in the obese group (*p* < 0.001). Sociodemographic characteristics of normal weight, overweight and obese groups are shown in Table 1. No statistical differences were detected between the groups in terms of sociodemographic data (*p* > 0.05).

**Table 1:** Socio-demographic characteristics of the groups.

	Normal weight	Overweight	Obese	*p*
Age	40.09 ± 7.26	41.20 ± 7.65	43.31 ± 7.88	0.148
Work any job, *n* (%)	23 (53.5)	17 (42.5)	13 (31.7)	0.131
Marital status, *n* (%)				
Married	27 (62.8)	28 (70.0)	32 (78.0)	0.31
Single	10 (23.3)	5 (12.5)	6 (14.6)	
Widowed/divorced	6 (13.9)	7 (17.5)	3 (7.4)	
Education, *n* (%)				
Primary education or less	22 (51.1)	23 (57.5)	28 (68.3)	0.143
High school	6 (14.0)	9 (22.5)	8 (19.5)	
University or higher	15 (34.9)	8 (20.0)	5 (12.2)	

**Note:**

*n*, number.

On comparing the clinical parameters between the three groups; significant differences were observed with respect to the results of the VAS, TPC, FIQ and HAM-D (*p* < 0.05). However, no significant difference was detected between groups for the results of HAM-A (*p* = 0.328). The highest scores were found in the obese group. Data are reported in Table 2.

**Table 2:** Clinical characteristics of the groups.

	Normal weight (*n* = 43)		Overweight (*n* = 40)		Obese (*n* = 41)		*p*
	median	min–max	median	min–max	median	min–max	
VAS	7	3–10	7	5–10	8	5–10	0.016
TPC	12	11–16	13	11–15	14	11–18	<0.001
FIQ	48	29–77	52.5	29–75	58	33–76	0.017
HAM-A	14	6–25	14	10–32	16	9–25	0.328
HAM-D	12	7–26	13	9–27	16	8–23	0.001

**Note**:

VAS, visual analog scale; TPC, tender point count; FIQ, fibromyalgia impact questionnaire; HAM-A, Hamilton anxiety scale; HAM-D, Hamilton depression scale; *n*, number; min, minimum; max, maximum.

Significant and positive correlations were found between BMI and VAS, TPC, FIQ and HAM-D scores in FMS patients (*r* = 0.277, *p* = 0.002; *r* = 0.384, *p* < 0.001; *r* = 0.292, *p* = 0.001; *r* = 0.357, *p* < 0.001, respectively). No significant correlation was detected between BMI and HAM-A (*r* = 0.164, *p* = 0.068) (Table 3).

**Table 3:** Correlations of clinical data with body mass index.

	*r*	*p*
VAS	0.277	0.002
TPC	0.384	<0.001
FIQ	0.292	0.001
HAM-A	0.164	0.068
HAM-D	0.357	<0.001

**Note:**

VAS, visual analog scale; TPC, tender point count; FIQ, fibromyalgia impact questionnaire; HAM-A, Hamilton anxiety scale; HAM-D, Hamilton depression scale.

## Discussion

We aimed to explain the associations between BMI and pain, TPC, disease activity, anxiety and depression in FMS patients.

Our study suggested that obese FMS patients had higher pain levels and TPC. This finding is consistent with prior research that has shown that severely obese FMS patients have higher pain scores and TPC (Kim et al., 2012). Further, Timmerman, Calfa & Stuifbergen (2013) reported positive correlations between BMI and pain and tender point index. Similarly, Neumann et al. (2008) demonstrated a negative correlation between BMI and tenderness threshold and a positive correlation between BMI and TPC. Aparicio et al. (2013) reported that both overweight and obese FMS patients have higher levels of pain than FMS patients with normal weight, but suggested that BMI is not significantly correlated with tenderness and TPC. Przekop et al. (2010) found an association between BMI and perceived pain in FMS patients. Yunus, Arslan & Aldag (2002) analyzed FMS patients classifying them into two groups based on their BMI levels, and found no significant difference between groups in terms of pain scores. Additionally, after adjustment for age and education, no significant positive correlation was found between BMI and TPC in their study. In agreement with the finding by Yunus, Arslan & Aldag (2002), Gota, Kaouk & Wilke (2015) reported no difference in pain scores among normal weight, overweight and obese FMS patients.

Disease activity—which was assessed by FIQ in our study—was positively correlated with BMI. This finding is consistent with that reported by Aparicio et al. (2013), where significant and positive correlations were reported between BMI and FIQ-subscales. Likewise, Kim et al. (2012) found a significant difference in terms of FIQ among the groups formed according to BMI levels. Vincent et al. (2014) also reported a positive correlation between BMI and FIQ. In contrast, no significant differences were detected among normal weight, overweight and obese groups in terms of FIQ by Gota, Kaouk & Wilke (2015). Additionally, Okifuji et al. (2010) did not detect an association between BMI and FIQ scores.

We found a positive correlation between BMI and depression and no significant correlation between BMI and anxiety. Kim et al. (2012) also found a significant difference between groups in terms of depression, and reported no significant difference in terms of anxiety. Further, Aparicio et al. (2013) reported that patients with a higher BMI had higher levels of depression and anxiety. Similarly, Senna et al. (2012) evaluated weight reduction in obese patients with FMS and reported that patients with weight-loss had better depression scores. On the contrary, Gota, Kaouk & Wilke (2015), Yunus, Arslan & Aldag (2002) and Okifuji et al. (2010) did not find any association between BMI and depression and anxiety.

There may be several explanations for the differences in the results mentioned above. Sample sizes and ethnicity may have played a role in the differences. Differences in the patient characteristics such as average BMI level, medication use, physical fitness, comorbidities, disease duration and education level may also influence the results. The clinical severity of the participants in our study which was evaluated by FIQ was found to be relatively lower than that in other relevant studies in the literature (Kim et al., 2012; Aparicio et al., 2013; Gota, Kaouk & Wilke, 2015). Medication use, physical activity level and sample size may have influenced the FIQ scores in our study. Therefore, our results may not be extrapolated to FMS patients with higher disease activity. In the abovementioned studies, researchers evaluated patients with various self-reported scales. Differences in these scales may have also affected the results.

It has not been fully understood how obesity influences the symptoms of FMS (Ursini, Naty & Grembiale, 2011). FMS is one of the main causes of disability and fatigue (Schweiger et al., 2017). FMS patients have reduced physical activity and exercise (Busch et al., 2011). This may explain why obesity occurs in FMS. Higher levels of proinflammatory cytokines have been determined in both FMS and obese patients (Catalán et al., 2007; Wang et al., 2008). Proinflammatory cytokines elevate pain and hyperalgesia (Zhang & An, 2007). Therefore, proinflammatory cytokines may also play a role in FMS and obesity association. Hypothalamic-pituitary-adrenal axis dysregulation was determined in both obese and FMS patients (Rosmond, Dallman & Björntorp, 1998). Roane & Porter (1986) indicated disturbances in the endogenous opioid system, which is important for pain regulation in hyperphagic obese Zucker rats. Obese patients have higher number of pain receptors in the fat tissue (Neumann et al., 2008).

Our study suggests that obese female FMS patients have higher levels of pain, TPC, disease activity and depression. Additionally, BMI was significantly and positively associated with the clinical manifestations of FMS. Therefore, FMS treatment programs should comprise weight loss strategies, life style modifications and a proper diet regimen. Medical or behavioral weight loss strategies should be used for overweight or obese FMS patients. One of the main problems in FMS is fatigue (Bartley, Robinson & Staud, 2018). Weight management strategies should consider the fatigue levels of FMS patients.

There are some limitations in our study. The sample size was small. We could not analyze differences regarding obesity grades because of the sample size. We defined obesity by calculating BMI. We did not use anthropometric measurements. We did not include male FMS patients. Medication use, physical activity level and fatigue level were not questioned. Most of the assessments were based on self-reported scales, which may affect their reliability. Our study did not have a prospective design. Therefore, we could not examine how weight loss would influence pain, TPC, disease activity, depression and anxiety. Future studies should comprise male FMS patients, and a control group without FMS, use anthropometric measurements for patients, and consider medication use, physical fitness levels, comorbidities and dietary habits of patients. Future prospective controlled studies may provide data on the causal relationship between BMI and severity of FMS symptoms.

## Conclusion

Obesity and being overweight are common in female FMS patients. Higher BMI levels are associated with clinical manifestations of FMS. Our study emphasizes the importance of maintaining a normal body weight in FMS patients. Future studies are needed to determine how FMS patients will respond to weight loss programs and which variables interfere with weight management in FMS patients.

## Supplemental Information

10.7717/peerj.4917/supp-1 Supplemental Information 1 Revised raw data.

10.7717/peerj.4917/supp-2 Supplemental Information 2 Patient questionnaire.

10.7717/peerj.4917/supp-3 Supplemental Information 3 Visual analogue scale.

10.7717/peerj.4917/supp-4 Supplemental Information 4 Fibromyalgia impact questionnaire.

10.7717/peerj.4917/supp-5 Supplemental Information 5 Hamilton depression scale.

10.7717/peerj.4917/supp-6 Supplemental Information 6 Hamilton anxiety scale.

## Additional Information and Declarations

### Data Availability

The following information was supplied regarding data availability: The raw data are provided in a Supplemental File.
