# Supplementary material for: The relationship between body mass index and pain, disease activity, depression and anxiety in women with fibromyalgia
Source: PeerJ. 2018 May 28;6:e4917. doi: 10.7717/peerj.4917 (PMC5978395; doi:10.7717/peerj.4917)
Supplement: Supplemental Information 2 [file peerj-06-4917-s002.docx]

**KAHRAMANMARAŞ SÜTÇÜ İMAM UNIVERSITY FACULTY OF MEDICINE**

**The Relationship Between Body Mass Index and Pain, Disease Activity, Depression and Anxiety in Women with Fibromyalgia**

**Name - Sirname:**

**How old are you?............**

**What is your marital status?**

1. **Single**
2. **Married**
3. **Widowed/divorcee**

**What is your educational status**

1. **Primary education or less**
2. **High school**
3. **University or higher**

**Work any job**

1. **Yes**
2. **No**

**Body mass index (height and weight measurements):**
