# Supplementary material for: The relationship between body mass index and pain, disease activity, depression and anxiety in women with fibromyalgia
Source: PeerJ. 2018 May 28;6:e4917. doi: 10.7717/peerj.4917 (PMC5978395; doi:10.7717/peerj.4917)
Supplement: Supplemental Information 3 [file peerj-06-4917-s003.pdf]

PAIN VISUAL ANALOGUE SCALE
& NUMERICAL PAIN RATING SCALE

VISUAL ANALOG SCALE (VAS) – (line is 10 cm long)

No pain | Pain as bad as it could be

No pain

Pain as bad as it could be

NUMERICAL PAIN RATING SCALE (NPRS)

0 1 2 3 4 5 6 7 8 9 10

No pain | Pain as bad as it could be
