# Supplementary material for: The relationship between body mass index and pain, disease activity, depression and anxiety in women with fibromyalgia
Source: PeerJ. 2018 May 28;6:e4917. doi: 10.7717/peerj.4917 (PMC5978395; doi:10.7717/peerj.4917)
Supplement: Supplemental Information 4 [file peerj-06-4917-s004.pdf]

FIBROMYALGIA IMPACT QUESTIONNAIRE (FIQ)

Name: ________________________________ Date: / /

Directions: For questions 1 through 11, please circle the number that best describes how you did overall for the *past week*. If you don't normally do something that is asked, cross the question out.

	Always	Most	Occasionally	Never
**Were you able to:**				
Do shopping? ……………………………	*0*	*1*	*2*	*3*
Do laundry with a washer and dryer?	*0*	*1*	*2*	*3*
Prepare meals?	*0*	*1*	*2*	*3*
Wash dishes/cooking utensils by hand?.....	*0*	*1*	*2*	*3*
Vacuum a rug?...	*0*	*1*	*2*	*3*
Make beds?	*0*	*1*	*2*	*3*
Walk several blocks?	*0*	*1*	*2*	*3*
Visit friends or relatives?	*0*	*1*	*2*	*3*
Do yard work?..	*0*	*1*	*2*	*3*
Drive a car?	*0*	*1*	*2*	*3*
Climb stairs?	*0*	*1*	*2*	*3*

12. Of the 7 days in the past week, how many days did you feel good?

0 1 2 3 4 5 6 7

13. How many days last week did you miss work, including housework, because of fibromyalgia?

0 1 2 3 4 5 6 7

(continued)

FIBROMYALGIA IMPACT QUESTIONNAIRE (FIQ) – page 2

Directions: For the remaining items, mark the point on the line that best indicates how you felt overall for the past week.

14. When you worked, how much did pain or other symptoms of your fibromyalgia interfere with your ability to do your work, including housework?

No problem with work ●___I ___I___I ___I___I ___I ___I ___I ___I___● Great difficulty with work

15. How bad has your pain been?

No pain ●___I ___I___I ___I___I ___I ___I ___I ___I___● Very severe pain

16. How tired have you been?

No tiredness ●___I ___I___I ___I___I ___I ___I ___I ___I___● Very tired

17. How have you felt when you get up in the morning?

Awoke well rested ●___I ___I___I ___I___I ___I ___I ___I ___I___● Awoke very tired

18. How bad has your stiffness been?

No stiffness ●___I ___I___I ___I___I ___I ___I ___I ___I___● Very stiff

19. How nervous or anxious have you felt?

Not anxious ●___I ___I___I ___I___I ___I ___I ___I ___I___● Very anxious

20. How depressed or blue have you felt?

Not depressed ●___I ___I___I ___I___I ___I ___I ___I ___I___● Very depressed

Fibromyalgia Impact Questionnaire (FIQ): Description and Scoring

The FIQ is an assessment and evaluation instrument developed to measure fibromyalgia (FM) patient status, progress and outcomes. It has been designed to measure the components of health status that are believed to be most affected by FM.

Content

The FIQ is composed of 10 items. The first item contains 11 questions related to physical functioning – each question is rated on a 4 point Likert type scale. Items 2 and 3 ask the patient to mark the number of days they felt well and the number of days they were unable to work (including housework) because of fibromyalgia symptoms. Items 4 through 10 are horizontal linear scales marked in 10 increments on which the patient rates work difficulty, pain, fatigue, morning tiredness, stiffness, anxiety and depression.

Administration

The FIQ is a self administered instrument that takes approximately 5 minutes to complete. The directions are simple and the scoring is self-explanatory.

Scoring

The FIQ is scored in such a way that a higher score indicates a greater impact of the syndrome on the person. Each of the 10 items has a maximum possible score of 10. Thus the maximum possible score is 100. The average FM patient scores about 50, severely afflicted patients are usually 70 plus. The questionnaire is scored in the following manner:

1. The first item consists of 11 questions that make up a physical functioning scale. The 11 questions are scored and summed to yield one physical impairment score. Each item is rated on a 4 point Likert type scale. Raw scores on each item can range from 0 (always) to 3 (never) - thus the highest total possible raw score is 33. Because some patients may not do some of the tasks listed, they are given the option of deleting items from scoring. In order to obtain a valid summed score for questions 1 through 11, the scores for the items that the patient has rated are summed and divided by the number of items rated (e.g. if the patient completed only 9 items at a score of 2 for each, the final score would be 9x2/9= 2). An average raw score between 0 and 3 is obtained in this manner.

2. Item 2 is scored inversely - so that a higher number indicates impairment (i.e., 0=7, 1=6, 2=5, 3=4, 4=3, 5=2, 6=1 and 7=0, etc.). Raw scores can range from 0 to 7.

3. Item 3 is scored directly (i.e. 7=7 and 0=0). Raw scores can range from 0 to 7.

4. Items 4 through 10 are scored in 10 increments .Raw scores can range from 0 to 10. 1f the patient marks the space between two vertical lines on any item, that item is given a score that includes 0.5.

5. Once the initial scoring has been completed, the resulting scores are subjected to a normalization procedure so that all scores are expressed in similar units. The range of normalized scores is 0 to 10 with 0 indicating no impairment and 10 indicating maximum impairment.

Scale	Item #	Recode	Score Range	Normalization
Physical impairment	1	No	0 - 3	S X 3.33
Feel good	2	Yes	0 - 7	S X 1.43
Work missed	3	No	0 - 7	S X 1.43
Do work	4	No	0 - 10	None
Pain	5	No	0 - 10	None
Fatigue	6	No	0 - 10	None
Rested	7	No	0 - 10	None
Stiffness	8	No	0 - 10	None
Anxiety	9	No	0 - 10	None
Depression	10	No	0 - 10	None

In order to maintain a maximum possible score of 100 it is necessary to employ an “equalization calculation” if a patient does not answer all 10 items. If one or more items are missed, the final summative score needs to by multiplied by 10/**x**. (e.g. if one question is missed multiply by 10/9 [i.e. 1.111], if 2 questions are missed multiply by 10/8 [i.e. 1.25 etc.])

Addendum
When the first version of the FIQ was developed, patients who were not working outside the home, were asked to skip the 2 questions regarding work. Therefore, a total score was made from the remaining 8 items. Since the revision of 1997 (unpublished), the work items have included housework so that all patients could potentially answer the work questions. Researchers over the years have used either 8 items or 10 items to form the total score. Users of the FIQ should indicate in their publications whether they used the 8-item method of deriving a total score or all 10 items. If they use the 8 item version they should multiply the total FIQ score by 10/8 (i.e. 1.25) so that results can be compared across studies.

Translations
The FIQ has been translated into at least 16 languages of which we are aware. These include: Swedish, Norwegian, Icelandic, Danish, Portuguese (Brazil, Portugal), Hebrew, Spanish (Spain, Mexico, Argentina, Cuba), German, Farsi, Arabic and French (France and Canada),Greek, Italian, Korean, Dutch and Turkish. Most of these translations have been validated.

FIQ *Citation*
Burckhardt, C.S., Clark, S.R, & Bennett, RM. (1991). The Fibromyalgia Impact Questionnaire: Development and validation. Journal of Rheumatology, 18, 728-734
