# Supplementary material for: The relationship between body mass index and pain, disease activity, depression and anxiety in women with fibromyalgia
Source: PeerJ. 2018 May 28;6:e4917. doi: 10.7717/peerj.4917 (PMC5978395; doi:10.7717/peerj.4917)
Supplement: Supplemental Information 5 [file peerj-06-4917-s005.pdf]

Hamilton Depression Rating Scale (HDRS)

Reference: **Hamilton M. A rating scale for depression. J Neurol Neurosurg Psychiatry 1960; 23:56–62**

Rating Clinician-rated

Administration time 20–30 minutes

Main purpose To assess severity of, and change in, depressive symptoms

Population Adults

Commentary

The HDRS (also known as the Ham-D) is the most widely used clinician-administered depression assessment scale. The original version contains 17 items ($HDRS_{17}$) pertaining to symptoms of depression experienced over the past week. Although the scale was designed for completion after an unstructured clinical interview, there are now semi-structured interview guides available. The HDRS was originally developed for hospital inpatients, thus the emphasis on melancholic and physical symptoms of depression. A later 21-item version ($HDRS_{21}$) included 4 items intended to subtype the depression, but which are sometimes, incorrectly, used to rate severity. A limitation of the HDRS is that atypical symptoms of depression (e.g., hypersomnia, hyperphagia) are not assessed (see SIGH-SAD, page 55).

Scoring

Method for scoring varies by version. For the $HDRS_{17}$, a score of 0–7 is generally accepted to be within the normal range (or in clinical remission), while a score of 20 or higher (indicating at least moderate severity) is usually required for entry into a clinical trial.

Versions

The scale has been translated into a number of languages including French, German, Italian, Thai, and Turkish. As well, there is an Interactive Voice Response version (IVR), a Seasonal Affective Disorder version (SIGH-SAD, see page 55), and a Structured Interview Version (HDS-SIV). Numerous versions with varying lengths include the HDRS17, HDRS21, HDRS29, HDRS8, HDRS6, HDRS24, and HDRS7 (see page 30).

Additional references

Hamilton M. Development of a rating scale for primary depressive illness. Br J Soc Clin Psychol 1967; 6(4):278–96.

Williams JB. A structured interview guide for the Hamilton Depression Rating Scale. Arch Gen Psychiatry 1988; 45(8):742–7.

Address for correspondence

The HDRS is in the public domain.

Hamilton Depression Rating Scale (HDRS)

PLEASE COMPLETE THE SCALE BASED ON A STRUCTURED INTERVIEW

Instructions: for each item select the one "cue" which best characterizes the patient. Be sure to record the answers in the appropriate spaces (positions 0 through 4).

1 DEPRESSED MOOD *(sadness, hopeless, helpless, worthless)*

- 0 |__| Absent.
- 1 |__| These feeling states indicated only on questioning.
- 2 |__| These feeling states spontaneously reported verbally.
- 3 |__| Communicates feeling states non-verbally, i.e. through facial expression, posture, voice and tendency to weep.
- 4 |__| Patient reports virtually only these feeling states in his/her spontaneous verbal and non-verbal communication.

2 FEELINGS OF GUILT

- 0 |__| Absent.
- 1 |__| Self reproach, feels he/she has let people down.
- 2 |__| Ideas of guilt or rumination over past errors or sinful deeds.
- 3 |__| Present illness is a punishment. Delusions of guilt.
- 4 |__| Hears accusatory or denunciatory voices and/or experiences threatening visual hallucinations.

3 SUICIDE
- 0 |__| Absent.
- 1 |__| Feels life is not worth living.
- 2 |__| Wishes he/she were dead or any thoughts of possible death to self.
- 3 |__| Ideas or gestures of suicide.
- 4 |__| Attempts at suicide (any serious attempt rate 4).

4 INSOMNIA: EARLY IN THE NIGHT
- 0 |__| No difficulty falling asleep.
- 1 |__| Complains of occasional difficulty falling asleep, i.e. more than ½ hour.
- 2 |__| Complains of nightly difficulty falling asleep.

5 INSOMNIA: MIDDLE OF THE NIGHT
- 0 |__| No difficulty.
- 1 |__| Patient complains of being restless and disturbed during the night.
- 2 |__| Waking during the night – any getting out of bed rates 2 (except for purposes of voiding).

6 INSOMNIA: EARLY HOURS OF THE MORNING
- 0 |__| No difficulty.
- 1 |__| Waking in early hours of the morning but goes back to sleep.
- 2 |__| Unable to fall asleep again if he/she gets out of bed.

7 WORK AND ACTIVITIES
- 0 |__| No difficulty.
- 1 |__| Thoughts and feelings of incapacity, fatigue or weakness related to activities, work or hobbies.
- 2 |__| Loss of interest in activity, hobbies or work – either directly reported by the patient or indirect in listlessness, indecision and vacillation (feels he/she has to push self to work or activities).
- 3 |__| Decrease in actual time spent in activities or decrease in productivity. Rate 3 if the patient does not spend at least three hours a day in activities (job or hobbies) excluding routine chores.
- 4 |__| Stopped working because of present illness. Rate 4 if patient engages in no activities except routine chores, or if patient fails to perform routine chores unassisted.

8 RETARDATION (slowness of thought and speech, impaired ability to concentrate, decreased motor activity)
- 0 |__| Normal speech and thought.
- 1 |__| Slight retardation during the interview.
- 2 |__| Obvious retardation during the interview.
- 3 |__| Interview difficult.
- 4 |__| Complete stupor.

9 AGITATION
- 0 |__| None.
- 1 |__| Fidgetiness.
- 2 |__| Playing with hands, hair, etc.
- 3 |__| Moving about, can't sit still.
- 4 |__| Hand wringing, nail biting, hair-pulling, biting of lips.

10 ANXIETY PSYCHIC
- 0 |__| No difficulty.
- 1 |__| Subjective tension and irritability.
- 2 |__| Worrying about minor matters.
- 3 |__| Apprehensive attitude apparent in face or speech.
- 4 |__| Fears expressed without questioning.

11 ANXIETY SOMATIC (physiological concomitants of anxiety) such as:
gastro-intestinal – dry mouth, wind, indigestion, diarrhea, cramps, belching
cardio-vascular – palpitations, headaches
respiratory – hyperventilation, sighing
urinary frequency
sweating
- 0 |__| Absent.
- 1 |__| Mild.
- 2 |__| Moderate.
- 3 |__| Severe.
- 4 |__| Incapacitating.

12 SOMATIC SYMPTOMS GASTRO-INTESTINAL
- 0 |__| None.
- 1 |__| Loss of appetite but eating without staff encouragement. Heavy feelings in abdomen.
- 2 |__| Difficulty eating without staff urging. Requests or requires laxatives or medication for bowels or medication for gastro-intestinal symptoms.

13 GENERAL SOMATIC SYMPTOMS
- 0 |__| None.
- 1 |__| Heaviness in limbs, back or head. Backaches, headaches, muscle aches. Loss of energy and fatigability.
- 2 |__| Any clear-cut symptom rates 2.

14 GENITAL SYMPTOMS (symptoms such as loss of libido, menstrual disturbances)
- 0 |__| Absent.
- 1 |__| Mild.
- 2 |__| Severe.

15 HYPOCHONDRIASIS
- 0 |__| Not present.
- 1 |__| Self-absorption (bodily).
- 2 |__| Preoccupation with health.
- 3 |__| Frequent complaints, requests for help, etc.
- 4 |__| Hypochondriacal delusions.

16 LOSS OF WEIGHT *(RATE EITHER a OR b)*

**a) According to the patient:**	**b) According to weekly measurements:**
0 |__| No weight loss.	0 |__| Less than 1 lb weight loss in week.
1 |__| Probable weight loss associated with present illness.	1 |__| Greater than 1 lb weight loss in week.
2 |__| Definite (according to patient) weight loss.	2 |__| Greater than 2 lb weight loss in week.
3 |__| Not assessed.	3 |__| Not assessed.

17 INSIGHT
- 0 |__| Acknowledges being depressed and ill.
- 1 |__| Acknowledges illness but attributes cause to bad food, climate, overwork, virus, need for rest, etc.
- 2 |__| Denies being ill at all.

Total score: |__|__|

This scale is in the public domain.
